# Evaluation of a Novel Fluorescent Marker for Continuous Live Imaging of Preimplantation Embryos

**DOI:** 10.3390/jdb14030039

**Published:** 2026-09-02

**Authors:** Debbie Montjean, Karine Audette, Simon Mathien, Marie-Hélène Godin-Pagé, Moncef Benkhalifa, Ibrahim Bilem, Pierre Miron

**Affiliations:** 1 Centres d’aide Médicale à la Procréation Fertilys, 1950 rue Maurice-Gauvin Street, #103, Laval, QC H7S 1Z5, Canada; 2 Institut de Recherche en Immunologie et en Cancérologie, Université de Montréal, 2950 Chemin de Polytechnique, Montréal, QC H3T 1J4, Canada; 3 Médecine et Biologie de la Reproduction et Laboratoire PERITOX, Université Picardie Jules Verne, CBH-CHU Amiens Picardie, 1 Rond-Point du Professeur Christian Cabrol, 80054 Amiens, France; 4 Saguaro Biosciences, La Centrale–Espace Entrepreneurial, Université Laval, Quebec, QC G1V 0A6, Canada

**Keywords:** embryo development, kinetic live imaging, live cell painting, fluorescent marker

## Abstract

This study evaluates ChromaLIVE^TM^, a multichromatic, mix-and-read fluorescent marker developed for continuous live-cell imaging, in the context of embryology. The aim was to evaluate whether this marker is compatible with dynamic embryonic development up to the blastocyst stage, with minimal impact on embryo viability, integrity, or normal growth of mouse embryos at the 2-cell stage that were cultured to the blastocyst stage under time-lapse imaging conditions, either in the presence or absence of ChromaLIVE^TM^. Results showed comparable blastocyst formation rates between ChromaLive^TM^ and the control group, as well as similar proportions of high-quality blastocysts. Although minor but statistically significant delays were noted in two kinetics events (t3 and tSB), other developmental timings remained unaffected. Time-lapse confocal fluorescence imaging demonstrated that ChromaLIVE^TM^ dye provided stable, bright, high-resolution 3D visualizations of cellular compartments including membranes, cytoplasm and embryonic structures such as trophectoderm, inner cell mass and blastocoel throughout development. Finally, transcriptomic analyses revealed a limited set of gene-expression differences between stained and unstained embryos; although these included developmental and metabolic pathways, embryos developed to the blastocyst stage comparably to controls.

## 1. Introduction

Research in embryology is evolving rapidly with the integration of computer vision and AI-driven analysis approaches, creating an increasing need for live-cell imaging markers [1,2,3,4,5,6,7]. Ideal markers must enable temporal tracking of cellular movement and divisions throughout development up to the blastocyst stage, without interfering with embryo culture conditions or compromising viability, integrity, and normal development [8,9]. Such markers must be biologically inert and non-toxic, inducing no cellular stress, developmental delays, apoptosis, or impaired growth. Additionally, they should be sensitive to environmental and physiological changes, allowing the detection of cellular dynamics in real time [10,11,12]. For long-term imaging, markers must also yield a stable, bright and quantifiable signal throughout extended culture periods, while minimizing phototoxicity [9,12,13]. However, the widespread adoption of live imaging techniques in routine research workflows has been constrained by the limited availability of fluorescent markers suitable for long-term imaging. Most commercially available markers are cytotoxic, require complex staining protocols or cannot visualize multiple cellular structures simultaneously, restricting the depth and quality of biological information captured [10,14,15,16]. Our study introduces ChromaLIVE^TM^, a non-target-specific, multichromatic live-cell dye that operates via a molecular rotor mechanism and passively integrates cellular membrane structures, enabling simultaneous visualization of multiple cellular organelles without requiring fixation or washing steps [13]. Several studies have subsequently demonstrated its compatibility with prolonged live-cell imaging in diverse cellular and 3D models, including human mesenchymal stem cells, primary chronic lymphocytic leukemia cells, cancer cell lines and liver spheroids [12,17,18,19,20,21]. These studies have shown that ChromaLIVE™ can provide temporally resolved information associated with changes in cellular morphology and physiological state.

These properties provided a rationale for investigating whether ChromaLIVE™ could be extended to preimplantation embryo imaging. However, because embryonic systems may respond differently to fluorescent probes than conventional cell or organoid models, the present study focused on evaluating its compatibility, safety and effectiveness in capturing key developmental dynamics during prolonged culture.

## 2. Materials and Methods

### 2.1. Mouse Embryos Culture Conditions

Cryopreserved 2-cell stage F1 hybrid mouse embryos (B6C3F1 × B6D2F1) were purchased for this study (Embryotech™ Laboratories Inc., Haverhill, MA, USA) and thawed following the provider’s recommendations. This F1 hybrid cross is the standard model used for embryo-based reagent testing in clinical embryology laboratories. The 2-cell stage mouse embryos were cultured to the blastocyst stage in SAGE 1-Step™ (CooperSurgical, Trumbull, CT, USA), containing ChromaLIVE™ (Saguaro Biosciences, Quebec, QC, Canada; 1:1000 dilution, which is the pre-validated working solution, *n* = 49) or in SAGE 1-Step™ medium alone, serving as the control group (Ctrl, *n* = 47). These embryos were maintained in an incubator equipped with Time-Lapse technology (MIRI^®^ TL, Esco Medical, Ramuciai, Lithuania; 37 °C, 5% CO_2_, 5% O_2_).

### 2.2. Morphokinetic Analysis

Embryonic development was continuously monitored using time-lapse imaging from the 2-cell stage to the blastocyst stage. Embryo quality was assessed morphologically following standard grading criteria, in which blastocyst quality and cell survival are evaluated visually. Developmental events were annotated and embryos were graded by a senior embryologist according to ESHRE guidelines [22]. Blastocyst rate was calculated as the number of blastocysts divided by the total number of embryos cultured. Embryo quality was assessed using the Gardner classification, with good-quality blastocysts defined as grade A or B [23,24].

### 2.3. Confocal Live Imaging Setting

A total of 19 live embryos were cultured in 35 mm uncoated Petri dishes (Cat. P35G-1.5–20-C, MAtTEK, Ashland, MA, USA) and imaged using the Yokogawa CellVoyager CQ1 (Yokogawa Electric Corporation, Tokyo, Japan) spinning disk confocal microscope equipped with an Olympus (Hachioji, Tokyo, Japan) UPLXAPO 20x dry lens (NA 0.75) and CellPathfinder software (version R3.08.03). Imaging was performed over a 96 h culture period to monitor live embryo development. High-resolution z-stack images were acquired every 6 h, with a 5 µm z-step between optical sections, for a total of 17 z-slices per embryo. Kinetic imaging intervals were timed to capture key development stages throughout the culture. Fluorescence signals from the 3 ChromaLIVE™ channels were collected, with excitation and emission settings listed in Table 1. Briefly, the first excitation wavelength was set at 488 nm, with fluorescence emission collected around 600 nm (ChromaLIVE-488_Yellow) and 700 nm (ChromaLIVE-488_Red). The second excitation wavelength was set at 561 nm, with fluorescence emission collected around 600 nm (ChromaLIVE-561).

### 2.4. Transcriptomic Analysis

Two-cell mouse embryos were cultured up to 96 h to the blastocyst stage under 3 different conditions: ChromaLIVE ^TM^ (1:1000, *n* = 3), DMSO (0.1% *v*/*v*, *n* = 3) and Control (no additive, *n* = 3). To distinguish dye-specific effects from vehicle effects, we used a two-factor design that accounted for the presence of solvent (DMSO). This approach allowed us to isolate dye-specific transcriptional responses independently of vehicle-related effects. RNA isolation from individual blastocysts cultured under each condition was performed using the SMART-Seq mRNA kit (Takara, Kusatsu, Shiga, Japan), following the manufacturer’s recommendations. Libraries were prepared as poly-A-selected, single-read (SR100) libraries and sequenced on an Illumina NextSeq 2000 (San Diego, CA, USA). The integrity of the raw sequencing data was assessed, with all FASTQ files meeting stringent base quality control standards (mean Phred score ≥ 30). Adapter sequences and low-quality bases were successfully removed using fastp, ensuring no significant overrepresented sequences remained post-trimming. Comprehensive quality reports generated by MultiQC confirmed uniform read quality and processing consistency across all samples. Transcript quantification was performed using Salmon, providing highly accurate expression estimates. This methodology accounted for fragment-level GC bias and sequence-specific biases, enhancing the reliability of the abundance estimates.

#### Differential Expression Analysis

Differential expression analysis was conducted with DESeq2 v1.40.1. Genes with an adjusted *p*-value (FDR) < 0.05 (Benjamini–Hochberg) and |log_2_FC| > 1 were deemed as differentially expressed genes (DEGs). Log fold changes were shrunk using lfcShrink with the type = “apeglm” or “normal” method as appropriate. Principal Component Analysis (PCA) was performed using rlog-transformed counts from DESeq2. Volcano plots and bar plots of DEGs were generated to illustrate the differential expression landscape between conditions.

### 2.5. Statistical Analyses

Statistical analyses were performed using the chi-square test for comparisons of rates and the Welch two-sample t-test for comparisons of continuous variables (ChromaLIVE™ group as compared to the Ctrl group). *p*-value < 0.05 was considered statistically significant.

## 3. Results

### 3.1. Blastocyst Assessment and Embryo Developmental Kinetics

The blastocyst rate (ChromaLIVE^TM^ = 73.4% vs. Ctrl = 82.9%) and good quality blastocyst (ChromaLIVE^TM^ =48.6% vs. Ctrl = 47.4%) rates were comparable between the two groups. ChromaLIVE^TM^ embryos reached certain developmental stages later than control embryos: t3 (ChromaLIVE^TM^ = 8.7 h vs. Ctrl= 6.8 h) and tSB (ChromaLIVE^TM^ = 59.3 h vs. Ctrl = 51.1 h) (Table 2). Importantly, other developmental timings were comparable in the presence of ChromaLIVE^TM^, with no difference in the cell cycles (s2, s3, cc3a, cc3b, cc3, ECC3) (Table 2 and Table 3).

### 3.2. Confocal Live Imaging

**Figure 1:**
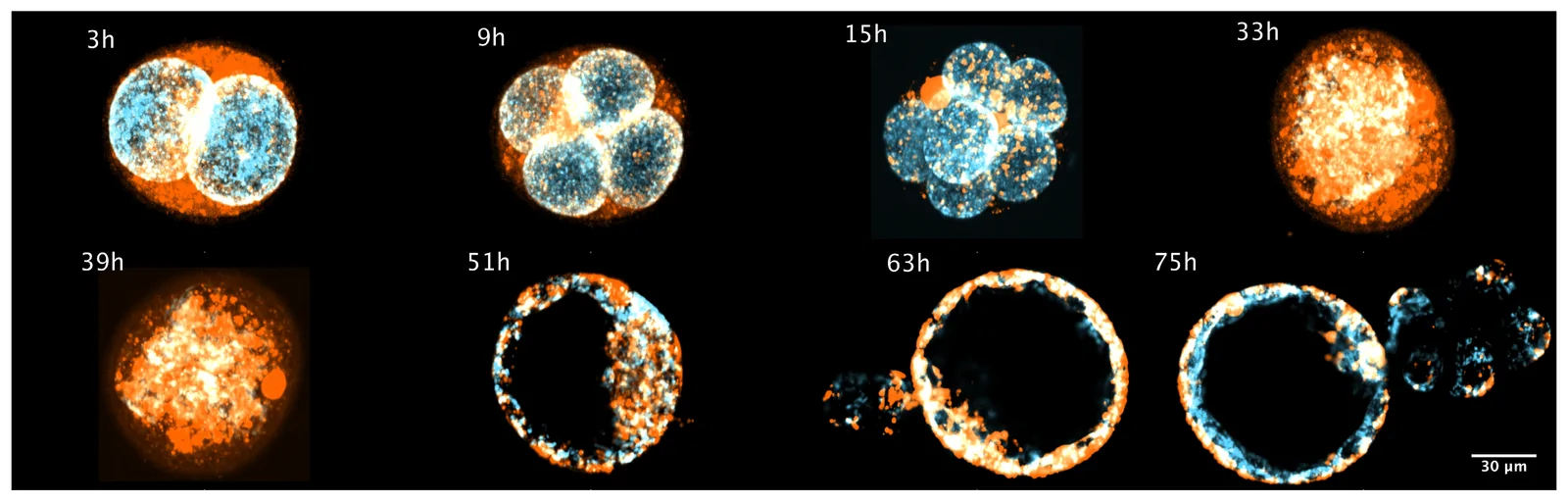
Kinetic imaging of ChromaLIVE^TM^-stained embryos showing temporal changes in staining patterns across development stages. Each panel depicts a maximum-intensity projection of merged ChromaLIVE^TM^ channels, with ChromaLIVE^TM^_488 displayed in orange and ChromaLIVE^TM^_561 in blue. *n* = 19 embryos.

The staining pattern of ChromaLIVE^TM^ clearly revealed well-distinguished embryonic compartments across developmental stages. During early cleavage stages (2-cell to 8-cell), blastomere membranes, cytoplasm and nuclei were distinctly visualized, while at the blastocyst stage, the trophectoderm, inner cell mass, and blastocoel cavity were readily distinguishable (Figure 1). This enabled detailed and dynamic 3D visualization of the embryo’s structural organization throughout development. A video of the ChromaLIVE^TM^ embryo development is available in the Supplementary Files.

### 3.3. Transcript Analyses

#### 3.3.1. Sequencing and Mapping Metrics

RNA-seq generated high-quality libraries across all conditions (control, DMSO, and ChromaLIVE^TM^). After preprocessing, reads mapped at high rates to the reference transcriptome (approximately 90% across samples), with >9 million reads quantified per sample. Gene-level summarization yielded an expression matrix of 77,179 genes across the cohort.

#### 3.3.2. Principal Component Analysis (PCA)

**Figure 2:**
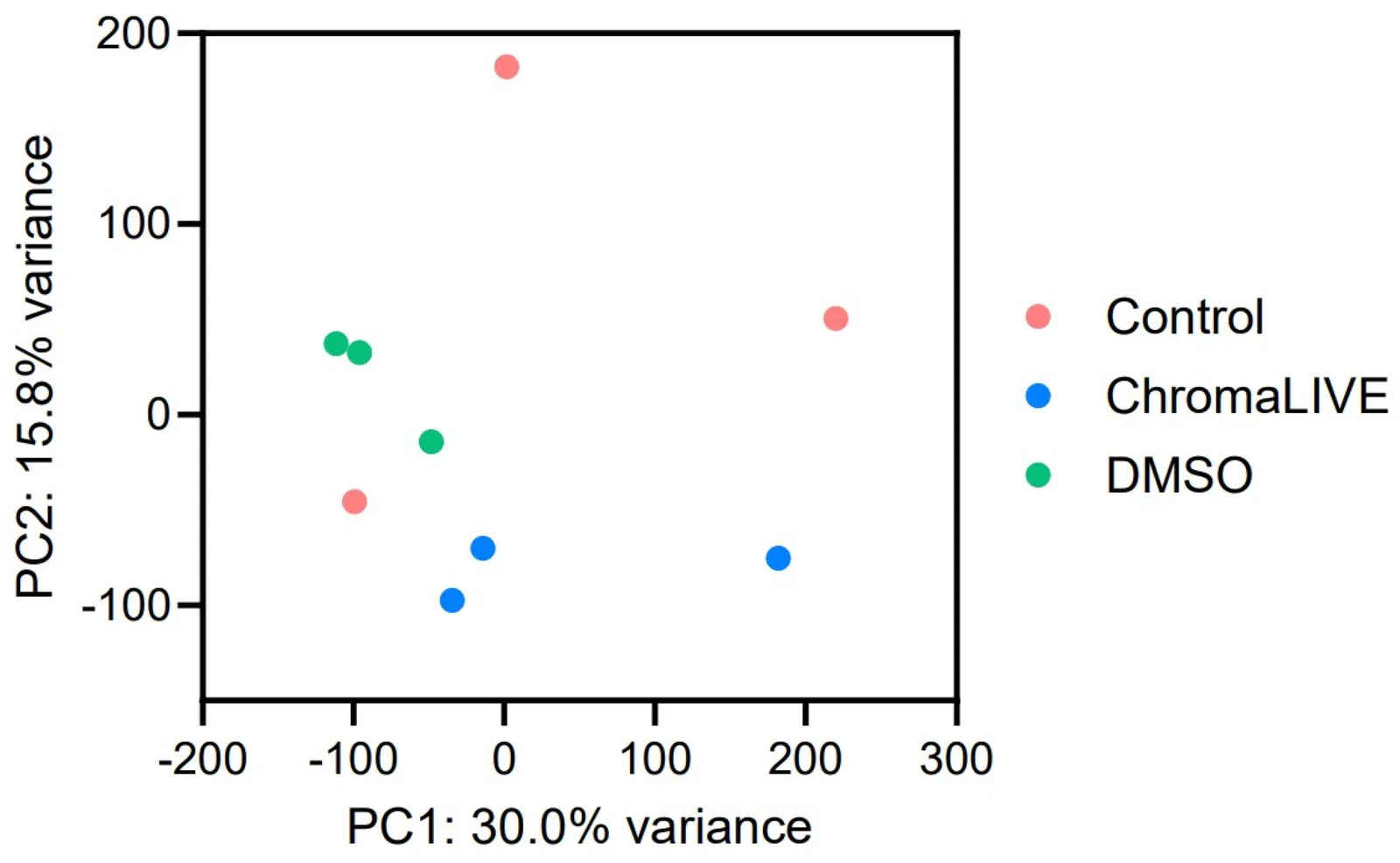
Principal Component Analysis of gene expression profiles. PCA was performed on variance-stabilized counts from all samples. The first two principal components are shown, explaining 30.0% and 15.8% of the total variance, respectively. *n* = 3 embryos per condition.

PCA was performed to evaluate global variance in gene expression across experimental groups (Figure 2). The distribution of samples in PCA space did not reveal distinct clustering associated with treatment condition. In particular, the first principal component (PC1), which explains approximately 30.0% of the total variance, did not separate ChromaLIVE™-treated, DMSO, and control samples. Overall, substantial overlap was observed among all groups across the first two principal components (PC1: 30.0%; PC2: 15.8%). Notably, greater dispersion was observed among control replicates along PC2 compared with ChromaLIVE™ and DMSO groups.

#### 3.3.3. Differential Expression Results

**Figure 3:**
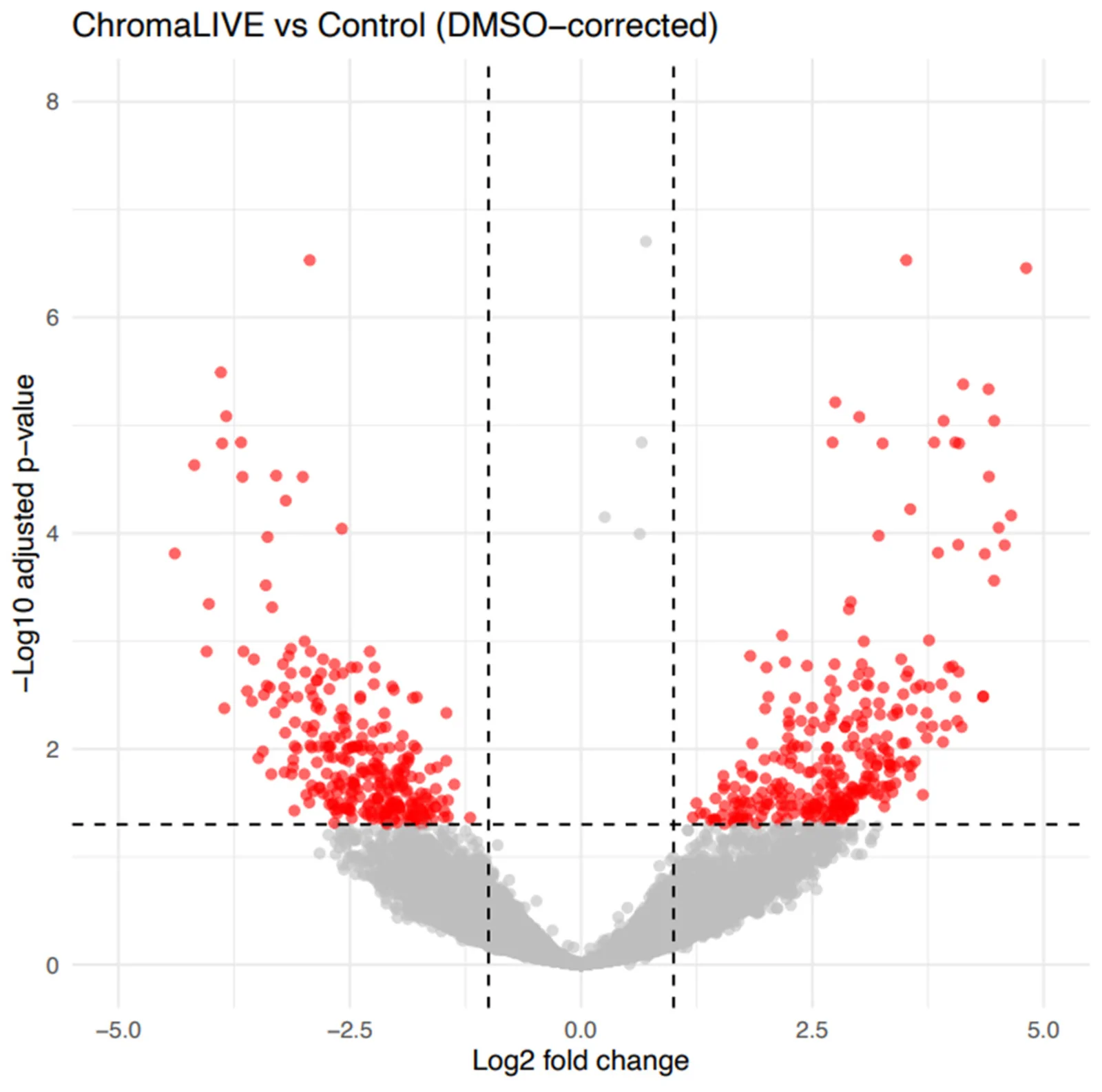
Volcano plot of differential gene expression in ChromaLIVE^TM^–stained embryos. The volcano plot displays DEGs in ChromaLIVE^TM^ condition compared to the Ctrl after correction for the DMSO solvent effect. The x-axis corresponds to the log_2_ fold change (log_2_FC), and the y-axis represents the −log_10_ adjusted *p*-value (FDR: False Discovery Rate). Red dots indicate DEGs meeting both statistical and biological thresholds (FDR < 0.05 and |log_2_FC| > 1), and gray dots represent genes that are not significantly differentially expressed (ns). Vertical dashed lines mark log_2_FC cutoffs, and the horizontal dashed line indicates the significance threshold (FDR = 0.05).

Consistent with the PCA results, differential expression analysis revealed a limited set of transcriptional changes associated with ChromaLIVE™ exposure. A total of 556 genes met differential expression criteria (FDR < 0.05 and |log_2_FC| > 1), representing approximately 0.72% of the expressed transcriptome (556/77,179 genes). Among these, 285 genes were upregulated and 271 were downregulated. While these represent a small fraction of detected features, they include genes associated with developmental and metabolic processes (Figure 3).

**Figure 4:**
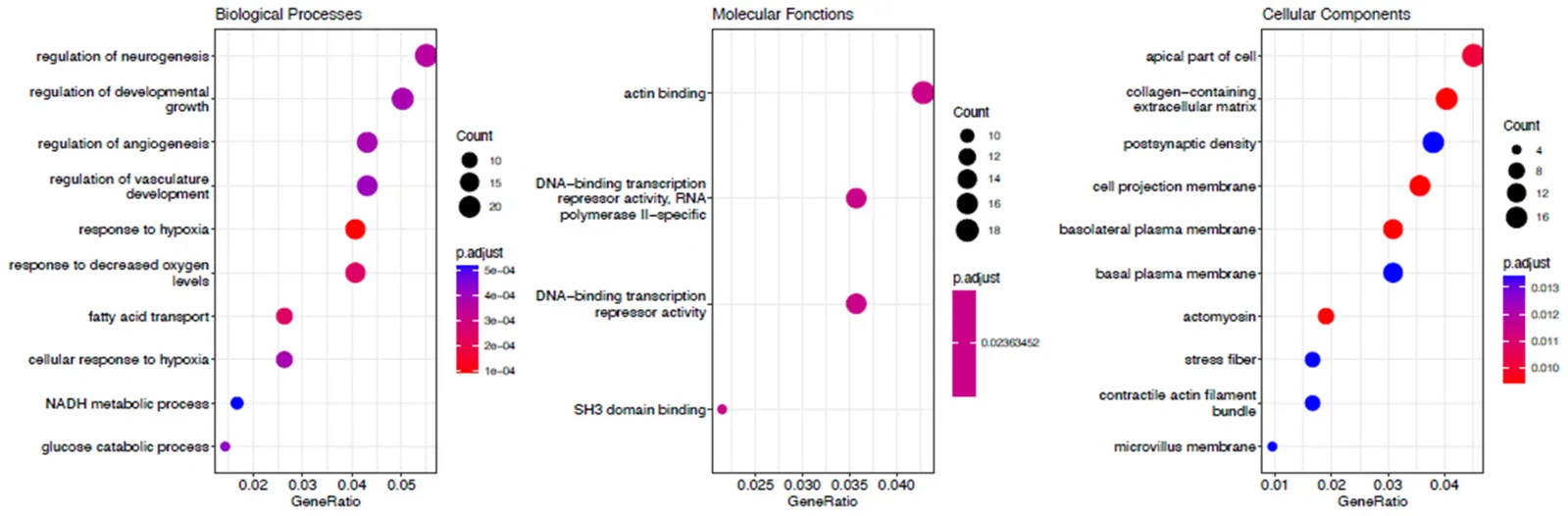
Gene Ontology enrichment analysis of differentially expressed genes in ChromaLIVE™-treated samples. Dot plots represent significantly enriched GO terms among DEGs (FDR < 0.05, |log_2_FC| > 1) for the three main ontologies: Biological Processes (left), Molecular Functions (center), and Cellular Components (right). Dot size indicates the number of genes associated with each term, while color reflects the adjusted *p*-value (Benjamini–Hochberg).

Among the most significantly upregulated genes were *Itgb3*, *Slc2a1*, *Sema4c*, *Sbsn*, and *Prlr*, representing transcripts involved in cellular adhesion, glucose transport, developmental signaling, epithelial differentiation, and hormone-responsive pathways (Figure 4). Conversely, strongly downregulated genes included *Txnip*, *Mrs2*, and *Npr3*, which participate in redox regulation, mitochondrial function, and signaling pathways (Figure 4). Importantly, these genes span diverse biological processes rather than clustering within a single functional pathway or stress-response program (Figure 4).

Enrichment analysis indicated that differentially expressed genes were associated with developmental growth, hypoxia, vasculature development, and metabolism (Figure 4). Canonical apoptosis- and stress-response transcripts were not prominently represented.

## 4. Discussion

In this study, we evaluated the effect of ChromaLIVE™ exposure on preimplantation mouse embryo development and morphokinetics in a time-lapse live-cell imaging culture system. Overall, embryos cultured in the presence of ChromaLIVE™ demonstrated comparable blastocyst formation rates and blastocyst quality to those cultured under standard conditions. These findings indicate that live embryos staining with this fluorescent probe over an extended culture period does not impair their ability to reach the blastocyst stage nor compromise morphological quality. Similar observations of preserved developmental competence following exposure to low-toxicity live-cell dyes have been reported in other mammalian embryo and stem-cell models [25,26,27,28].

Despite these comparable global outcomes, differences in early developmental kinetics were observed. While most developmental stages were unaffected, embryos exposed to ChromaLIVE exhibited delayed timing at early cleavage events (t3) and onset of blastulation (tSB). In contrast, other cleavage events from t4 to stages associated with compaction (tSC and tEC) occurred within a timeframe comparable to controls, indicating preservation of the overall development kinetics.

The observed delays may reflect a transient cellular adaptation to the presence of the dye, rather than toxic effects. Potential contributing factors may include metabolic adaptations or alterations in redox homeostasis. An additional factor that could have contributed to the kinetic delay is the solvent of ChromaLIVE™ dye. The working solution contains 0.1% DMSO, which may affect developmental timing [29,30]. To isolate dye-specific transcriptional responses from vehicle-related effects, our transcriptomic analysis included a DMSO-only control group. While DMSO alone induced minimal transcriptional changes, the combination of dye and solvent may underlie the observed transient kinetic delay. The vehicle was further controlled in the transcriptomic experiment, which revealed a DMSO-specific effect on development. The early-cleavage delays (t3) resolved by the blastocyst stage are consistent with a transient rather than sustained effect.

From a regulatory perspective, these early kinetic differences are inconsequential because they did not impair developmental competence. The two-cell mouse embryo assay (MEA) is a non-binding guidance developed to detect embryotoxic contaminants in culture media and reagents [31,32]; the present study was not designed as a formal MEA compliance assessment, and this specific threshold is therefore not used here as a pass/fail criterion. The blastocyst formation rate obtained for the ChromaLIVE™ group (73.4%) falls within the broader range of acceptance limits (≥70–80%) employed in contemporary quality-control practice and peer-reviewed literature [33], and the two groups did not differ significantly in blastocyst formation. Moreover, the absence of significant differences in later time points, including t4 to stages associated with compaction, suggests that embryos are able to compensate for early kinetic delays. This developmental plasticity aligns with existing evidence that mammalian embryos can recover from moderate stressors, as long as critical cellular thresholds are not exceeded [34,35,36]. Preimplantation embryos are particularly sensitive to environmental perturbations during early cleavage, when epigenetic reprogramming and metabolic shifts occur [37]. However, these shifts did not persist into later milestones in our study.

At the molecular level, transcriptomic profiling of ChromaLIVE™-exposed embryos, corrected for the DMSO solvent effect, identified 556 differentially expressed genes (285 upregulated, 271 down-regulated), representing less than 1% of the expressed transcriptome. The limited proportion of affected transcripts, together with the balanced distribution of up- and down-regulated genes, indicates that ChromaLIVE™ exposure induces modest transcriptional changes. Interestingly, the differentially expressed genes do not belong to signaling pathways associated with cellular viability, embryonic development, or other critical processes responsible for embryo integrity (Figure 4), thereby reinforcing the hypothesis that ChromaLIVE™ exerts no toxic effect. Importantly, global transcriptional profiles showed no distinct clustering of stained embryos compared with DMSO-only and unstained samples, while greater variability was observed among control replicates, suggesting that intrinsic biological heterogeneity outweighed any dye-related transcriptional effects. Collectively, these findings indicate that ChromaLIVE™ does not produce major transcriptomic perturbations under the conditions tested. Prior validation of the dye in other live-cell imaging studies using diverse cell types further supports its favorable safety profile [12,13,17,18,19,20,21].

Future studies should focus on optimizing the application of ChromaLIVE™ in embryology by refining imaging parameters such as exposure duration, excitation intensity, imaging frequency and evaluating formulations with reduced DMSO content to further minimize potential DMSO effects on highly sensitive cellular models. Adjustment of fluorescence imaging settings to minimize light exposure frequency has been shown to reduce phototoxic effects in gametes and embryos [38,39], and may further attenuate the observed early kinetic delays while preserving imaging quality. Investigating additional molecular and cellular responses, such as mitochondrial function, reactive oxygen species production, spindle integrity, and DNA-damage markers, would provide deeper insight into the cellular adaptations associated with kinetic live imaging. In addition, epigenetic analyses of embryos cultured with and without ChromaLIVE™ would determine whether transient kinetic shifts are associated with persistent changes in DNA methylation patterns relevant to post-implantation development.

Beyond demonstrating biocompatibility, our results also highlight the imaging performance and biological informativeness of ChromaLIVE™. Although the mechanisms underlying ChromaLIVE™ redistribution during embryo development remain to be established, the broad staining patterns observed throughout embryo development are consistent with the reported physicochemical properties of this dye [13]. Unlike conventional targeted probes that bind covalently to specific molecular targets or require enzymatic activation, ChromaLIVE™ operates via a molecular rotor mechanism and is highly sensitive to the viscosity and polarity of the local physicochemical environment. Consequently, changes in intracellular properties can result in changes in fluorescence intensity and staining pattern across its different spectral channels. This complementary information on cellular membranes and intracellular compartments may explain the ability of this dye to simultaneously visualize multiple embryonic structures. In the present study, the dye enabled consistent, high-resolution visualization of multiple cellular compartments throughout preimplantation development, including blastomere membranes and cytoplasm during early cleavage, as well as trophectoderm, inner cell mass, and blastocoel formation at the blastocyst stage. This multiplex structural resolution provides spatial and temporal information that is typically difficult to obtain using conventional live-cell dyes, which often label only single organelles or require complex staining procedures. Compared with label-free approaches such as phase-contrast or differential interference contrast (DIC) microscopy, ChromaLIVE™ resolves individual blastomeres and delineates membranes, cytoplasm, and specific structures in three dimensions and over time. This facilitates tasks that are difficult with label-free imaging alone, such as counting cells and tracking defined cellular structures. The ability to simultaneously resolve multiple embryonic compartments in a non-invasive manner enhances interpretability of developmental dynamics and may facilitate quantitative morphokinetic analyses and computational image-based phenotyping. Thus, ChromaLIVE™ offers substantial analytical value for studying embryo architecture and developmental processes in real time.

Several limitations of this study should be considered when interpreting these findings. The morphokinetic experiment did not include a dedicated DMSO-matched cohort; consequently, dye- and vehicle-specific effects on morphokinetic data cannot be fully disentangled from the present design. Embryo numbers, particularly for the transcriptomic analysis (*n* = 3 per condition), limit statistical power and should be considered when interpreting the differential expression results. Embryo quality was assessed morphologically rather than through quantitative live/dead cell counts, and confocal imaging was performed at 20× magnification, which, while sufficient to address the present research questions, does not resolve subcellular detail achievable at higher magnification. Finally, as noted above, the F1 hybrid strain used here develops more robustly in vitro than inbred strains, so the tolerance observed may not generalize without confirmation in additional genetic backgrounds.

Extending analyses beyond the blastocyst stage is essential to assess potential influence of ChromaLIVE™ on implantation success, early placentation, or fetal development. Longitudinal studies, including embryo transfer into pseudo-pregnant murine recipient females, are necessary to evaluate implantation rates and post-implantation outcomes. Because F1 hybrid embryos are more robust in vitro than inbred embryos, the developmental tolerance observed here may not fully generalize to inbred strains; confirmation in such strains would be a useful extension. Finally, as non-invasive embryo assessment through live-cell imaging technologies becomes increasingly integrated into assisted reproduction, standardized frameworks for safety validation of fluorescent probes across laboratories, culture systems, and imaging platforms will be essential [40,41]. In this context, the application of ChromaLIVE™ in AI-based embryology studies represents a promising future direction, offering high-quality and high-content time-resolved data to train predictive algorithms for embryo selection. Overall, our results indicate that, under the conditions tested, ChromaLIVE™ enables high-resolution live imaging for tracking embryo development with no evidence of a major impact on blastocyst formation or quality, while producing a limited transcriptional response. Nonetheless, careful interpretation and further validation remain essential when extending live-cell imaging assays beyond preimplantation stages.

## 5. Conclusions

This study highlights the feasibility of using ChromaLIVE™, a fluorescent marker for continuous tracking of embryos without impairing their development or blastocyst quality. Although slight differences in early cleavage kinetics were observed, RNAseq analysis evidenced that these variations reflect benign molecular and cellular adaptations, confirming the marker’s safety at both the phenotypic and molecular levels. These findings validate this marker as a robust tool for high-resolution embryo imaging, with potential applications in reproductive research and AI-driven embryo assessment.

## Figures and Tables

**Table 1:** Imaging parameters for the ChromaLIVE^TM^ dye.

Dye	Excitation (nm)	Emission (nm)	Exposure (ms)
ChromaLive 488_Yellow	488	617/73	500
CH3:ChromaLive 488_Red	488	685/40	500
CH4: ChromaLive_561	561	617/73	500

**Table 2:** Morphokinetic parameters in the ChromaLIVE ^TM^ as compared to the control group. tn: time to *n* number of blastomeres. tM: time to morula; tSC: time to start of compaction; tEC: time to end of compaction; tSB: time to start of blastulation. A Welch *t*-test was used to compare the two culture conditions. * *p* < 0.05. *n* = number of visualized and annotated events.

	ChromaLive™					Control					Welch *t*-Test		
	Mean	Median	Max	Min	*n*	Mean	Median	Max	Min	*n*	Qobs	*p*-Value:	95% Confidence Interval
t3	8.7	8.7	16.7	3.1	30	6.8	5.2	19.0	3.5	30	−2.21	0.03 *	[−3.78; −0.19]
t4	9.8	9.5	23.1	4.5	30	8.0	6.3	19.9	4.0	34	−1.81	0.07	[−3.69; 0.19]
t5	18.7	18.4	30.7	14.0	18	17.0	15.9	26.6	14.1	27	−1.44	0.15	[−4.12; 0.71]
t6	20.2	19.8	39.5	14.2	20	18.1	17.0	27.4	14.7	31	−1.48	0.14	[−4.93; 0.79]
t7	20.5	19.8	29.0	15.0	14	17.9	17.4	21.8	15.6	14	−2.07	0.05	[−5.15; 0.034]
t8	23.8	22.8	35.0	16.7	17	21.2	19.0	36.9	16.1	28	−1.66	0.1	[−5.82; 0.59]
t10	30.9	30.0	41.6	22.0	15	29.2	27.8	45.0	22.5	23	−0.95	0.35	[−5.139; 1.87]
tM	35.9	33.6	67.4	26.9	16	32.3	32.2	45.2	26.7	23	−1.34	0.19	[−9.13; 1.98]
tSC	39.4	35.3	70.6	29.5	18	34.8	33.9	46.7	28.5	23	−1.54	0.13	[−10.64; 1.55]
tEC	43.3	41.9	67.6	32.2	17	39.7	37.7	50.9	32.2	21	−1.28	0.21	[−9.42; 2.22]
tSB	59.3	56.8	86.2	43.6	21	51.1	49.7	70.0	41.5	24	−2.77	0.01 *	[−14.21; −2.19]

**Table 3:** Embryonic cell cycles in the ChromaLIVE ^TM^ group as compared to the control group. s2: time between division to three cells and subsequent division to four cells. s3: time between division to five cells and subsequent division to eight cells; cc3: blastomere cell cycle duration of the third cell cycle (a = t5 − t4, b = t6 − t4, c = t7 − t4); ECC3: embryo cell cycle (t8 − t4). A Welch *t*-test was used to compare the two culture conditions. No statistically significant difference was found. *n* = number of visualized and annotated events.

	ChromaLive™					Control					Welch *t*-Test		
	Mean	Median	Max	Min	*n*	Mean	Median	Max	Min	*n*	Qobs	*p*-Value:	95% Confidence Interval
s2	1.6	1.2	9.1	0.5	27	1.0	0.9	4.0	0.5	30	−1.61	0.1	[−1.22; 0.14]
s3	4.2	3.6	9.3	0.7	14	3.7	2.4	13.8	1.0	23	−0.52	0.60	[−2.61; 1.54]
cc3a	9.4	8.7	16.5	7.6	18	9.5	9.7	12.5	7.4	27	0.29	0.77	[−0.99; 1.33]
cc3b	10.7	9.6	16.7	8.1	20	10.4	10.3	13.5	8.0	31	−0.44	0.67	[−1.65; 1.07]
cc3c	12.3	11.2	19.2	8.4	14	11.4	11.0	15.7	8.7	14	−0.92	0.37	[−3.02; 1.16]
ECC3	14.9	13.9	28.8	9.0	17	13.2	12.1	21.8	9.0	28	−1.25	0.22	[−4.48; 1.09]

## Data Availability

Data Availability Statements are available internally.

## Supplementary Materials

The following supporting information can be downloaded at: https://www.mdpi.com/article/10.3390/jdb14030039/s1. Video S1: Video of a ChromaLIVE^TM^ embryo development.
